# Laparoscopic fertility-sparing surgery for early stage ovarian cancer: a single-centre case series and systematic literature review

**DOI:** 10.1186/1757-2215-7-59

**Published:** 2014-05-29

**Authors:** Antonella Cromi, Giorgio Bogani, Stefano Uccella, Jvan Casarin, Maurizio Serati, Fabio Ghezzi

**Affiliations:** 1 Gynecologic Oncology Unit, Department of Obstetrics and Gynecology, University of Insubria, Del Ponte Hospital, Piazza Biroldi 1, 21100 Varese, Italy

**Keywords:** Ovarian cancer, Laparoscopy, Fertility-sparing, Pregnancy, Staging

## Abstract

**Background:**

There is as yet limited evidence about fertility-sparing surgery for early ovarian cancer (EOC) carried out laparoscopically. We sought to analyze recurrence patterns and fertility outcome in a cohort of ovarian cancer patients who underwent fertility-saving laparoscopic surgical staging.

**Methods:**

We conducted a retrospective analysis of prospectively collected data on all patients undergoing fertility-sparing laparoscopic staging procedures for presumed EOC at a single gynecologic oncology service. Oncologic safety and reproductive outcome were the main outcome measures. The pertinent literature is reviewed.

**Results:**

The study cohort consisted of 12 women. Cases included 5 invasive epithelial tumors and 7 nonepithelial tumors. The disease was reclassified to a higher stage in one woman. After a median follow up period of 38 months (range: 14–108), the overall survival was 100% and recurrence-free survival 90.9%. Five (100%) of patients who attempted pregnancy conceived spontaneously. Three of them had uneventful term pregnancy delivering healthy babies. The literature search yielded 62 cases of laparoscopic fertility conserving surgery for ovarian cancer. There were 4 (6.2%) recurrences. Cumulative pregnancy and live birth rate were not estimable as earlier publications lack essential data.

**Conclusions:**

Laparoscopic staging may represent a viable option for premenopausal women seeking fertility preservation in the setting of early ovarian cancer. More research is needed to determine whether laparoscopy may offer reproductive benefits to this particular population.

## Background

While ovarian cancer is predominantly a disease of older, post-menopausal women, it is estimated that in western countries approximately 12% of cases will be diagnosed in women younger than 45 years of age [1]. Each year in Europe and in the US over 10,000 new cases of malignant ovarian neoplasms occur in women of the reproductive age group [1,2].

In today’s society, women often postpone childbearing for cultural, financial and professional reasons, and in their late 30s to early 40s are attempting to get pregnant for the first time. As a result, the demand for fertility-sparing options grows among young women with cancer, including ovarian cancer, which is still the deadliest of all gynecologic cancers. Younger women are more likely to be initially diagnosed with stage I disease [3], for which 5-year survival rates approach 90% [1], and gynecologic oncologists are increasingly facing the challenge of how to manage early-stage ovarian carcinoma in premenopausal women who desire future fertility.

Accumulated experience indicates that the oncologic results following conservative treatment with retention of the uterus and contralateral ovary are reassuring and similar to standard staging, and shows acceptable recurrence rates [4-6]. However, there are no randomized trials comparing the traditional comprehensive staging to fertility-sparing surgery and it is unlikely that such a trial will ever be conducted. Studies dealing with fertility-sparing treatment in the last 20 years are often limited by retrospective nature, small sample size, short or unknown follow-up periods, lack of stratification by histologic type and grading [5,6]. Evidence is even more scant and problematic for fertility-sparing procedures *c*arried out laparoscopically [7-12]. Unlike what occurred with endometrial and cervical cancer, the widespread acceptance and adoption of laparoscopic staging of early ovarian cancer (EOC) has been much slower and minimal access surgery in this context is still regarded as investigational, despite initial recognition of its role by leading oncology societies [13,14].

The advantages of a laparoscopic approach, including less tissue and organ handling, lower bleeding, and lower contamination with foreign bodies than open surgery, by reducing the potential for adhesions may be particularly beneficial in women wishing to preserve their fertility. Moreover, avoiding a midline abdominal scar from xiphoid to pubis, which can alter one’s perception of body image with a negative impact on self-esteem or self-confidence, may be especially important for young EOC patient [15,16].

As surgical pioneers began to accumulate sufficient data in support of minimally invasive techniques for EOC, cohort studies are emerging in which a few conservatively treated patients have been included [7-12,17-22]. However, absence of oncologic detriment and fertility outcome cannot be inferred due to small numbers, failure to analyze treatment effects within this subgroup of patients, commixture of low malignant potential and invasive tumors.

In this article, we report our experience with laparoscopic treatment of premenopausal women seeking fertility preservation in the setting of a unilateral ovary-confined ovarian cancer and we review currently available data regarding the results of fertility-sparing minimally invasive surgery for EOC.

## Methods

We have collected prospective data on all patients undergoing fertility-sparing laparoscopic staging procedures for presumed early-stage ovarian cancers from December 2004 to December 2012 at the Gynecologic Oncology Division of University of Insubria, Varese, Italy.

Laparoscopic approach for the staging of early stage ovarian cancer was introduced at our Department in January 2003. Once laparoscopic techniques have been incorporated in the management of apparent stage I ovarian cancers, from that moment onwards this approach was offered to each patient presenting with that condition, unless specific contraindications existed, such as a documented significant cardiopulmonary disease. No patient was refused laparoscopic surgery for reasons of tumor size, obesity, prior surgical history, anticipated difficulty of resection. In women less than 40 years of age who have not yet completed childbearing, the option of conservative treatment with retention of the uterus and contralateral ovary has been offered. A complete obstetric and gynecological history was obtained to identify patients with underlying potential infertility problems. Infertility work-up to document fertility potential was not required prior to the procedure. Women who were in the late 30’s were made aware that their fertility potential was obviously less.

Fertility-sparing laparoscopic surgery was offered as an alternative therapy with modifications of standard treatment, according to institutional guidelines that dictate the required elements of informed consent. We did not perform such procedures as a part of a formalized research program. The specific consent form is aimed at maximizing patient understanding of the non-standard nature of the fertility preserving treatment, and forces patients to weigh the risks and benefits associated with each treatment option. A very thorough and extensive counseling, highlighting the risks potentially inherent in non-standard care, has been given to the patient and partner, and immediate family members before the decision to proceed with a fertility sparing surgery. At our institution research activities involving the collection or study of existing data are exempt from the requirement of IRB approval*.*

For the purposes of this study*,* early ovarian cancer (EOC) was defined as an ovarian tumor grossly limited to one ovary, with no evidence of intraperitoneal disease. During the initial laparoscopic examination, patients who were found to have the disease beyond the ovary were excluded. The procedures were performed by surgeons with extensive training and experience in gynecologic oncology and in advanced minimally invasive surgery. Detailed description of surgical technique used for laparoscopic staging of EOC has been reported elsewhere [23].

The department’s contemporaneously collected surgical database was reviewed. All histologic types of adnexal cancers, including stromal and germ cell tumors were included. Intraoperative details and post-operative complications were prospectively collected within the surgical database at the end of the procedure and at discharge from hospital. The database is updated on a regular basis and contains detailed information on post-treatment follow*-*up*,* including reproductive outcomes data. Intraoperative mass rupture was defined as any rupture, intentional or unintentional, that resulted in spill of cyst contents into the peritoneal cavity. Operative times were defined as ‘skin-to-skin’ time. Any late (>30 days) surgical complications and follow-up information are systematically added to the database by the reviewing clinician. The first 7 patients of the study cohort had been included in previously published studies on laparoscopic staging of EOC [17,23].

### Literature review

A comprehensive PUBMED literature search was conducted searching from January 1995 to August 2013 and including only English language citations. The search headings “ovarian cancer”, “fertility-sparing”, and “laparoscopy” were used. Included were reports which addressed specifically laparoscopic fertility-sparing staging at first diagnosis of invasive ovarian or fallopian tube cancer.

We excluded series including borderline tumors if it was not possible to discriminate data related to women with invasive cancer, as the surgical aim for staging differed from that with invasive disease and follow up data would be skewed. Also excluded were series in which patients undergoing fertility-sparing surgery were non separable from other laparoscopically managed EOC patients.

## Results

A total of 12 patients underwent fertility-conserving laparoscopic surgery for presumed stage I ovarian cancer. Median age of the patients was 31 (range: 13–39) years, with 7/12 (58.3%) patients who were referred for restaging following cystectomy or salpingo-oophorectomy with findings of invasive disease on final pathology and 5/12 (41.7%) women presenting with adnexal masses suspicious for malignancy. Cases included 5/12 (41.7%) invasive epithelial tumors and 7/12 (58.3%) nonepithelial tumors. Tumor characteristics are outlined in Table 1.

**Table 1:** Hystologic types, tumor grading and stage after comprehensive surgical staging

	**Study cohort**
	**(N = 12)**
*Histotype*	
Endometrioid	2 (16.7%)
Serous	1 (8.3%)
Mucinous	1 (8.3%)
Undifferentiated carcinoma	1 (8.3%)
Dysgerminoma	2 (16.7%)
Granulosa cell tumor	2 (16.7%)
Immature teratoma	1 (8.3%)
Malignant struma ovarii	2 (16.7%)
*Grading*	
G1	4 (33.3%)
G2	2 (16.7%)
G4	1 (8.3%)
Rare histologic types (grading not performed)	5 (41.7%)
*Final stage*	
I a	5 (41.7%)
I c	5 (41.7%)
II b	1 (8.3%)
III c	1 (8.3%)

Data are expressed as number (%).

Intra- and post-operative details are provided in Table 2. No conversion to laparotomy and no intraoperative complication occurred. Five (41.7%) patients experienced intraoperative cyst rupture. A 23-year old girl developed one month after surgery a pleural empyema that resolved with appropriate antibiotic and did not result in patient morbidity.

**Table 2:** Intra- and postoperative details

	**Study cohort**
	**(N = 12)**
Operative time (min)	320 (120–450)
Blood loss (mL)	100 (10–500)
Blood transfusions	0
Pelvic lymphnodes (n)	21 (16–39)
Paraaortic lymphnodes (n)	7 (2–16)
Intraoperative complications	0
Postoperative complications	1 (8.3%)
Hospital stay (days)	3 (1–8)

Data are expressed as median (range) or number (%).

Based on final pathology, one (1/12, 8.3%) patient was upstaged to stage IIIC. Histology revealed an undifferentiated carcinoma and upstaging was due to para-aortic lymph node involvement. After thorough counseling the patient underwent a repeat operation to complete staging with hysterectomy and contralateral oophorectomy. She received first line chemotherapy with a combination of carboplatin and pacitaxel and a second-line regimen with doxorubicin due to evidence of progressive disease. She died of disease 11 months after diagnosis. This patient has been excluded from any subsequent analyses of outcome data.

Two patients with endometroid carcinoma and one with mucinous histology were given adjuvant chemotherapy with single agent carboplatin (AUC 6 mg/mL per min) for six cycles. One woman with a G2 serous carcinoma received a combination of carboplatin (AUC 6 mg/mL per min) and Paclitaxel (175 mg/m^2^) for six cycles.

Two women with struma ovarii were managed postoperatively with total thyroidectomy and radioactive I-131 ablation. Menstrual function was preserved in all patients. Five of 5 (100%) patients who attempted pregnancy conceived. One had a miscarriage at 12 gestational weeks; a 20-year old girl opted for termination of pregnancy due to social reasons; 3/5 (60%) patients had uncomplicated term pregnancies, two of which after receipt of chemotherapy, with no congenital anomalies in the offspring. None received an infertility treatment before pregnancy. A patient with serous epithelial ovarian cancer who achieved a term pregnancy was offered the option to complete the surgical staging but she opted to postpone the definite treatment after a second pregnancy will be obtained.

One patient with a granulosa cell tumor, who achieved pregnancy one year after primary procedure, developed pelvic peritoneal recurrence 27 months after diagnosis. She underwent recurrence resection, completion of staging, and chemotherapy with cisplatin/etoposide/bleomycin. She is currently disease free after 5-year follow-up.

After a median follow up period of 38 months since diagnosis (range: 14–108), the overall survival in our cohort was 100% and recurrence-free survival 90.9%.

### Literature review

**Figure 1:**
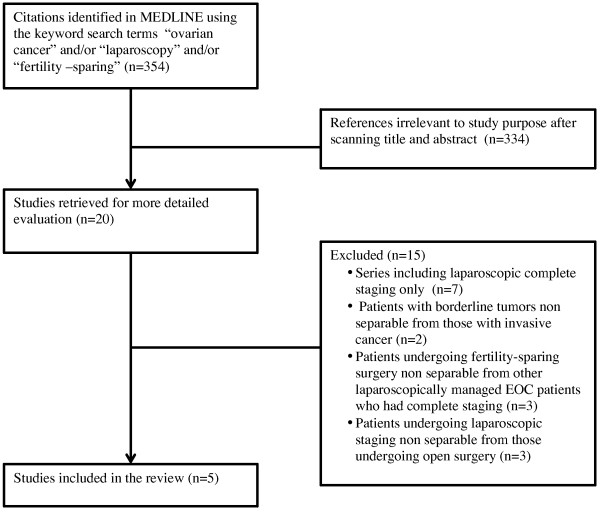
**Flowchart that summarizes the process and results of our literature review** ***.***

The PUBMED search identified 354 citations; 334 were excluded and 20 retrieved for detailed review (Figure 1). Following exclusions, see Figure 1 for reasons, there were five identified papers reporting oncologic and/or reproductive outcomes after fertility-sparing laparoscopy for ovarian cancer (Table 3). All previous reports are single institution case reports of, at most 17 women. Overall, there were 4/62 (6.2%) recurrences. Cumulative pregnancy and live birth rate were not estimable as earlier publications lack essential data.

**Table 3:** Laparoscopic fertility-sparing staging for presumed early stage adnexal cancers; systematic review of literature and current data

**Study**	**Data collection**	**Patients (n)**	**Epithelial histotype (n)**	**Recurrence (n)**	**Pregnancy (n)/women seeking pregnancy (n)**	**Follow-up (months)**
Leblanc [7]	Prospective	9	*	0	3/not reported	*
Muzii [9]	Prospective	15	11	1	3/not reported	*
Nezhat [10]	Retrospective	5	5	1	*	*
Cheng [11]	Retrospective	17	17	1	5/8	61 (17–115)
Brockbank [12]	Prospective	16	*	1	*	*
Current study	Prospective	11†	4	1	5/5	28 (7.3–101)
**Total**		**73**	**-**	**5 (6.8%)**	**-**	**-**

*Not possible to discriminate data related to women with EOC undergoing fertility sparing procedures from those who had comprehensive staging.

†One patient upstaged after intial fertility-sparing surgery has been excluded as she ultimately underwent a comprehensive staging.

## Discussion

The current study and our literature review revealed a small but growing body of evidence that fertility-saving laparoscopic surgical staging can be offered to selected women with EOC as a viable fertility-sparing alternative to traditional laparotomy.

Ovarian cancer is the most deadly of all malignancies of the female genital tract. In the past, survival, the primary goal of cancer treatment, tended to overshadow survivorship considerations. However, there has been a clear improvement in five-year survival for stage I patients since the late 1980s [24], and therefore, issues affecting long-term cancer survivors has become more widely recognized. In recent decades there has been an increased emphasis on tailoring treatment to provide fertility-sparing options without compromising oncologic outcomes in women who present with malignancy that appears confined to the ovary. However, fertility-sparing surgery results in a deliberately incomplete staging and therefore poses a major clinical challenge since this may potentially increase the risk of recurrence. In a series including 118 women with EOC that appeared to have disease confined to one ovary, after full staging the contralateral ovary had microscopic disease without spread elsewhere in 2.5% of cases [25].

Experience with open surgery indicates that the oncologic outcomes following fertility-sparing procedures are similar to those with complete surgical staging. A recent review of the published literature identified 15 studies with a total of 918 young women with EOC who underwent fertility-sparing surgery [6]. They had a 11.9% chance of recurrence and 5.2% chance of death from disease, which is comparable to fully-staged historical controls [26,27]. Combining the published literature, out of a total 918 women, there were 242 pregnancies from 177 women, resulting in 215 live births. Miscarriage rate was about 10%. Although these results are encouraging, series of more than 20 patients do show recurrent disease presenting confined to the preserved ovary in 4.8% of cases [4]. Moreover, available outcome data should be viewed in light of some limitations. First, there is a lack of prospective data. Second, it is not known whether the histological subtype, breast cancer gene status or other factors influence the prognosis for these patients with early stage disease when treated conservatively. Third, the appropriateness of offering fertility sparing procedures for grade 3 or stage IC tumors, or in selected cases with disease extended beyond the ovaries [28] is highly debated. Finally, in several series pregnancy rates are not calculated using women attempting to conceive as the denominator [29,30].

The type of surgical approach is a further variable to be taken into account in an already-complex field. A limited but expanding body of literature suggests that laparoscopic comprehensive staging of apparent EOC is technically feasible and can provide equivalent surgical assessment when compared with laparotomy. Minimally invasive surgery offers several established advantages to the cancer patient needing surgery: less bleeding, less trauma, less risk of incisional hernias and postoperative infection, and quicker, less painful recovery [31]. Besides these clearly defined advantages, laparoscopic staging may offer reproductive benefits to women seeking fertility preservation in the setting of ovarian carcinomas grossly confined to one ovary, but this is not proven. A laparoscopic approach has been indicated as preferable to laparotomy for fertility-sparing surgeries due to reduced adhesion formation following laparoscopy and avoidance of laparotomy, known to decrease fecundity [31]. Postsurgical adhesions may adversely affect fertility by distorting adnexal anatomy and interfering with gamete and embryo transport. To date, no comparative studies of laparoscopy vs. open surgery in women undergoing fertility-saving procedures for a gynecologic cancer have been published, so potential benefits on reproductive function are only inferred from multiple observations in non-oncological medical fields [32,33].

Furthermore, undefined risks of port site metastasis, tumor seeding and intraoperative tumor rupture associated with laparoscopic staging continues to be debated. Available data suggest that these risks may not outweigh the clearly established benefits of a minimally invasive access in the setting of careful patient selection and preoperative assessment. However, the safety issues become even more crucial in the context of fertility-sparing surgery that results in an intentionally incomplete staging. Hu et al. in a retrospective series of 94 women who had fertility-sparing treatment for EOC found that a laparoscopic approach did not affect overall and disease-free survival [21]. Kajiyama et al. [27] assessed survival after fertility-sparing surgery separately for women with stage Ic due to iatrogenic cyst rupture versus Ic due to malignant cells in peritoneal washings or surface involvement. They found no significant difference in DFS and OS between patients with “iatrogenic” stage IC and those with stage IA, whereas survival outcomes of the patients with “biologic” IC disease were poorer than those of patients with stage IA. Notwithstanding the lack of unequivocal evidence that cyst rupture in patients who would have been stage IA and who are upgraded to stage IC solely because of the occurrence of intraoperative rupture worsens the prognosis, this observation is helpful for the definition of selection criteria for the optimal candidate for laparoscopic fertility-sparing treatment for EOC. Indeed, whether stage IC tumors are suitable for fertility sparing surgery is debatable and guidelines diverge in their recommendations. Minimal access surgery is more likely than laparotomy to result in intentional capsular rupture, since large tumors often require drainage before removal both to allow specimen retrieval and to achieve adequate working space. It is hoped that future data will better address whether intentional capsular rupture during surgery should be distinguished from preoperative spillage of cyst contents in terms of potential to compromise the success of cancer therapy in young women undergoing laparoscopic surgical staging for EOC with fertility*-*sparing intent.

There have been few studies performed with the specific intent of reporting recurrence in patients who underwent a more conservative, fertility-sparing laparoscopic staging. In several series [17-22] oncologic outcomes of women undergoing fertility-sparing surgery are not described separately from those of the entire cohort, thus preventing any risk estimate. Given the rarity of early stage adnexal tumors in reproductive-age women, clinical decisions on the surgical approach in this population will need to be made from case series reports, of which this current series adds to the scant available literature. This study represents the first attempt to prospectively assess the results of laparoscopy in this specific group of EOC patients. Hopefully, as more and more cancer centers offer laparoscopic staging of EOC, data of the subpopulation of women undergoing fertility-sparing surgery will be reported separately.

A significant proportion of patients who do not try to get pregnant after fertility preserving procedures is a consistent finding in the published series on this issue [4]. For those patients with malignant germ cell tumors, the earlier mean age at time of diagnosis and prediction of good outcome after fertility preservation, are the most likely reasons for postponing childbearing. Women electing not to attempt pregnancy after conservative surgical management of ovarian carcinomas poses more complex issues. A strong desire on the part of the patient to retain fertility potential is a crucial point in the selection of patients for performing fertility sparing procedures. Young women should be made aware that, although there is no evidence that currently used fertility preservation options directly compromise the success of cancer therapy, the conventional accepted treatment remains comprehensive staging. Questions still remain on the acceptability of conservative options where fertility is not an immediate concern.

## Conclusion

Preliminary data are beginning to surface that demonstrate the efficacy of laparoscopic fertility-sparing staging of EOC, although sample size is too small and duration of follow-up is still too short to make definitive statements. More research is needed to determine whether laparoscopy affects the reproductive outcome and the oncologic safety of this deliberately incomplete staging in this specialized population. Only the results of future data collection will provide treating oncologists and cancer patients with valuable information to make specific decisions on fertility-sparing options.

## Competing interests

The authors declare that they have no competing interests.

## Authors’ contributions

AC wrote the manuscript and participated in the design of the study. GB helped to draft the manuscript and performed the literature review. SU discussed results, interpretation, and presentation. JC collected and analyzed the data*.* MS co-worked on associated data collection and their interpretation. FG conceived the study, participated in its design and revised it critically. All authors have contributed to, seen and approved the manuscript.
